# Arsenic Removal and Iron Recovery from Arsenic-Bearing Iron Ores by Calcification-Magnetic Roasting and Magnetic Separation Process

**DOI:** 10.3390/ma16216884

**Published:** 2023-10-26

**Authors:** Mengbo Dai, Yongcheng Zhou, Qingfei Xiao, Jinfang Lv, Lingyun Huang, Xian Xie, Yiming Hu, Xiong Tong, Tiejun Chun

**Affiliations:** 1 School of Metallurgical Engineering, Anhui University of Technology, Maanshan 243032, China; diamonb@126.com (M.D.); 13855515822@126.com (Y.H.); 2 Faculty of Land and Resource Engineering, Kunming University of Science and Technology, Kunming 650093, China; xiaoqf801002@163.com (Q.X.); jflv2017@126.com (J.L.); hly0510@126.com (L.H.); kgxianxie@126.com (X.X.); kgxiongtong@163.com (X.T.); 3 Yunnan Key Laboratory of Green Separation and Enrichment of Strategic Mineral Resources, Kunming 650093, China

**Keywords:** arsenic, iron ore, calcification-magnetic roasting, magnetic separation

## Abstract

Extracting iron while minimizing the health and environmental risks associated with arsenic contamination necessitates the removal of arsenic from arsenic-bearing iron ores to ensure a safe and sustainable supply of this metal for industries. The beneficiation of iron minerals and arsenic-bearing minerals from arsenic-bearing iron ores with a calcification-magnetizing roasting and low-intensity magnetic separation (CMR-LMS) process is investigated in this work. The results show that the process is successful in extracting iron minerals and eliminating arsenic-containing minerals. The roasting involves two key steps: calcification and magnetizing, which change hematite and goethite into magnetite and arsenic-bearing minerals into calcium arsenates. The process’s separation efficiency of the CMR-LMS is closely linked to the parameters such as roasting temperature, roasting time, coke, alkalinity, and the liberation of gangue minerals from iron minerals. Through grinding and secondary magnetic separation, the iron minerals and gangue components, as well as arsenic, in roasted sand can be further separated. The optimum procedure results in a high-grade iron concentrate with an iron assay of 65.65%, an Fe recovery rate of 80.07%, and an arsenic content of 0.085%, while achieving a 93.29% As removal rate from the original ore that has 45.32% Fe and 0.70% As.

## 1. Introduction

Arsenic is a common element in the Earth’s crust, with an average concentration of five parts per million [1,2]. So far, four major types (arsenides, sulphides, oxides, and arsenates) [1] and more than 300 kinds of arsenates and associated minerals have been discovered [3]. The most common arsenic minerals are arsenopyrite (FeAsS), enargite (Cu_3_AsS_4_), tennantite (Cu_12_As_4_S_13_), realgar (As_4_S_4_), and orpiment (As_2_S_3_) [4]. Arsenic is one of the most common harmful impurities in iron ores, with the common occurrences being arsenopyrite and its weathering products, such as scorodite (FeAsO_4_·2H_2_O). The mass fraction of arsenic in iron ores is generally low, usually below one percent.

Beneficiation of valuable metals from iron ores can be achieved through flotation or magnetic separation technologies, taking into account the difference in wettability or the coefficient of magnetization between arsenopyrite or other arsenic-bearing sulfide minerals and other iron minerals such as magnetite, hematite, and goethite [5,6,7]. However, it has been experimentally proved that it is difficult to effectively beneficiate the metal values from such iron ores via physical beneficiation methods, such as flotation or magnetic separation. Roasting [2,8,9,10,11,12,13,14,15], sintering [3,16,17,18,19,20], and other chemical beneficiation methods are the conventional pyrometallurgical routes of arsenic-bearing ores, due to the volatility of arsenic and many of its oxides. Although the roasting and sintering processes removed the arsenic, it is costly as it requires gas-cleaning facilities and produces highly toxic gas, which could result in severe environmental contamination.

With the continuous depletion of iron ores and the increasing demand for iron metals in the future, iron ore resources in China are becoming increasingly scarce. It is necessary to choose appropriate processes for removing arsenic in order to efficiently use arsenic-bearing minerals. There is a great quantity of iron ore with arsenic deposited in China, the reserves of which amount to 1.88 billion tons in the homeland as of 1990. In the Yunnan Province of China, many years of mining activities have left more than 30 million tons of arsenic-bearing iron ore, the main As-containing mineral species being ferric arsenate. The iron ore featured a fine dissemination of scorodite into weakly magnetic iron minerals such as goethite and hematite. The arsenic-bearing iron ores in China have become an important source for the production of iron products. However, the large-scale utilization of arsenic-containing iron ores is disappointingly scarce, due to insufficient methods available to handle such iron ores.

Our suggestion is to enhance the magnetization roasting process with an elevated alkalinity, and concurrently achieve arsenic solidification during the magnetization roasting. The calcification-magnetizing roasting and low-intensity magnetic separation (CMR-LMS) process is able to separate arsenic and iron minerals from the As-bearing iron ores by converting hematite and goethite to magnetite and ferric arsenate to calcium arsenate. The process of magnetizing roasting was used to reduce hematite and goethite to magnetite [21]. Calcium oxide has the ability to more effectively convert iron arsenate into more thermodynamically stable calcium arsenates, thus eliminating arsenic. The decomposition temperature of iron arsenate and calcium arsenates is above 1630 °C and 2740 °C, respectively, which implies that calcium arsenates are more thermodynamically stable than iron arsenates, thus providing a secure and long-term disposal form of arsenic at ambient pressure [22].

The purpose of this project is to transform the weakly magnetic iron minerals, such as scorodite, into magnetite and calcium arsenate via calcification-magnetizing roasting. Subsequently, a low-intensity magnetic separation is conducted to extract the iron minerals along with their associated gangue minerals, such as calcite, dolomite, and silicates. Due to an inadequate separation of the gangue components from the magnetic concentrate, the reported magnetic concentrate grades and iron recoveries are currently unsatisfactory. We propose to further refine the primary magnetic separation concentrate to maximize the dissociation of the gangue components and enhance the removal rate of As. The primary magnetic product is further ground and liberated to achieve a high-quality magnetic concentrate through the second LMS reconcentration. This work aims to investigate the feasibility of recovering weak magnetic iron minerals and eliminate arsenic from arsenic-bearing iron ores through CMR-LMS technology, in order to develop an economical method for iron extraction and arsenic removal from arsenic-bearing iron ore.

## 2. Materials and Methods

### 2.1. Materials

**Figure 1:**
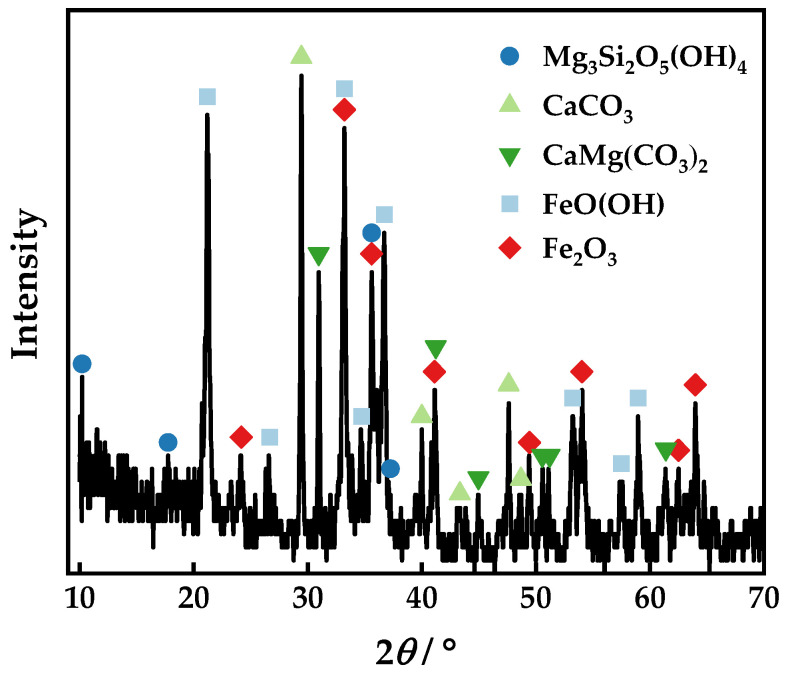
XRD pattern of the arsenic-bearing iron ore.

The arsenic-bearing iron ore was sourced from Yunnan Province. Samples for chemical analysis were collected through a process of multi-point sampling and division. Analysis of the sample revealed that the ore had 0.70% As and 45.32% Fe with a binary alkalinity of 1.24, as demonstrated in Table 1. As illustrated in Table 2, the iron present in the ore is mainly composed of hematite and limonite, with a minor proportion of magnetite. The other iron minerals were siderite, ferrosilite, and pyrite, in combination with C, Si, and S, respectively. The distribution of iron minerals indicates that the oxidation degree of iron is high, and the magnetic properties of the raw materials of the iron ore may be weak. High valent iron oxides were identified in the XRD (X-Ray diffraction) spectrum of the ore in Figure 1. The phase retrieval results demonstrate that the diffraction peak intensity of carbonate and hydroxide phases is relatively high, indicating that most of the losses in Table 1 are carbonate and hydroxide. As the concentration of arsenic-bearing minerals was lower than the XRD detection limit of 1%, it was not detected in the sample. The complex mineral phase and arsenic content indicate the difficult utilization characteristics of the mine.

**Figure 2:**
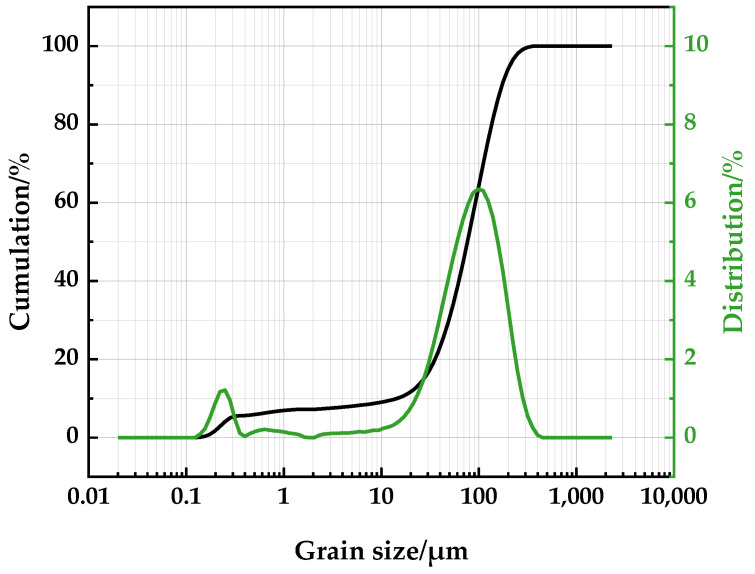
Particle size analysis of the arseniferous iron ore.

**Figure 3:**
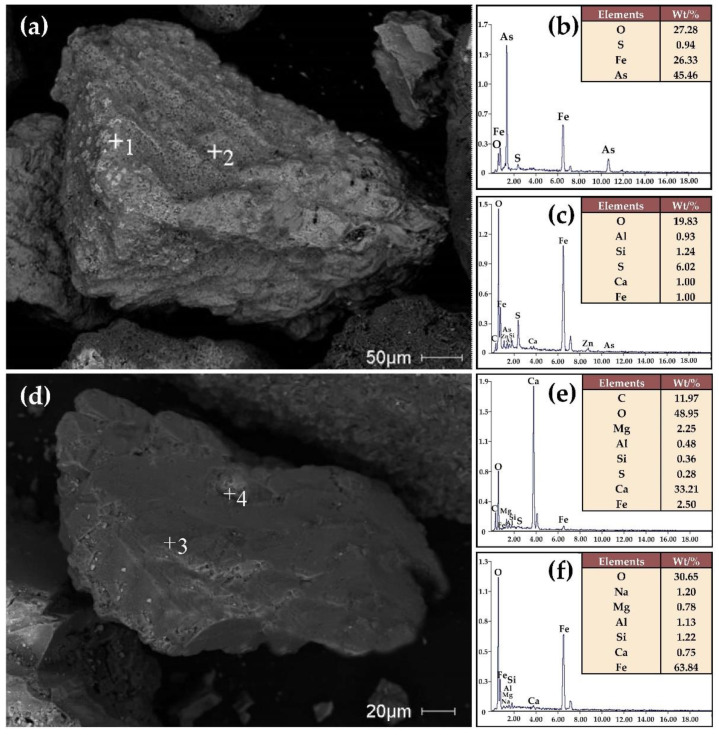
Microscopic morphology (**a**,**d**) of the arsenic-bearing iron ore and elements analysis of points 1–4 categorized as (**b**,**c**,**e**,**f**), respectively.

The iron ore containing arsenic has a certain level of fineness. Analysis (Figure 2) with the laser particle size analyzer revealed that the particle size of this mineral is mainly distributed between 0.139 and 0.448 μm and 10.59 and 399.4 μm, and the peak particle size of the mass fraction distribution is 105.4 μm. The true density of this ore is greater than the standard cement sample, which is 3.79 g/cm^3^, as shown in Table 3. According to the Blaine method [23], the specific surface area was measured to be 603.8 cm^2^/g, which is significantly lower than the standard cement sample, being only one-sixth of it. Figure 3 displays the ore sample that has been imaged using a combination of an SEM (scanning electron microscope) and EDX (energy dispersive X-ray detector). Microscopic observation revealed two particles of irregular shape and angular form. The largest observed sizes of the particles were 301 μm and 231 μm, respectively, which are within the expected range of particle sizes observed in laser particle size analysis. Analysis of the four randomly chosen points on the two particles reveals that the ore is mainly composed of oxides of iron and other metals, accompanied by a fraction of carbonates and silicate. The EDX analysis corroborates the chemical analysis results described in Table 2. It was apparent from the observation that it was complex to extract arsenic from the iron minerals through physical separation.

Coke fines used as a reductant in the calcification roasting were obtained from KunSteel Holding Co., Ltd., Kunming, China. The coke fines with a particle size less than 75 μm were obtained by sieving, with the proximate analysis of 82.34% fixed carbon, 2.38% volatile matters, 1.02% water, and 14.26% ash. Calcium oxide (purity 99%) was used to increase alkalinity and as a dearsenic agent in the calcification roasting, which was obtained from the Damao Chemical Reagent Factory, Tianjin.

### 2.2. Methods

#### 2.2.1. Technical Route for CMR-LMS

**Figure 4:**
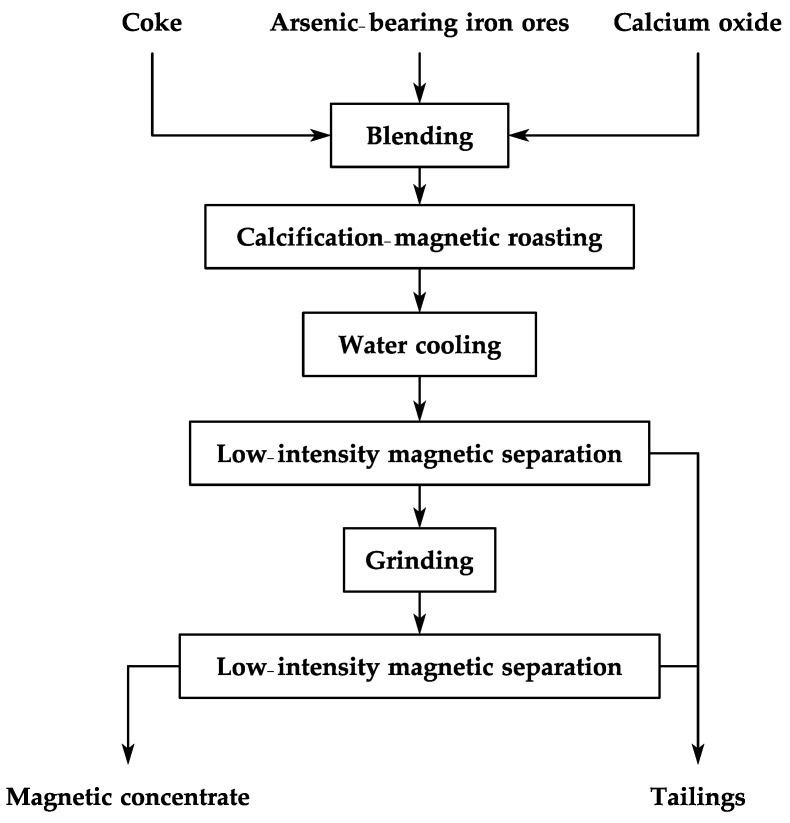
Schematic representation of the CMR-LMS process.

The CMR-LMS combined technology is used to select high-grade iron concentrate from arsenic-bearing iron ore. The flowsheet is shown in Figure 4. A specific proportion of calcium oxide, coke powder, and arsenic-bearing iron ore are blended together in an even manner. The mixture is then placed in a corundum crucible and heated in a Muffle furnace (MF-1700C, manufactured by Beiyike Equipment Technology Co., Ltd., Hefei, China) until the set temperature of 650–850 °C is attained. At the completion of the given roasting time (20–60 min), the roasted sample is swiftly removed and cooled with water. Utilizing a Davis tube (magnetic tube CXG-08SD, manufactured by Shida automation instrument technology Co., Ltd., Tangshan, China) at an intensity of 0.2 T, the cooled sample was separated, recovering the iron minerals and their associated gangue minerals such as calcite, dolomite, and quartz, resulting in a primary magnetic concentrate and a tailing. The primary magnetic product is then subjected to wet ball milling (XMQ-240 × 90, manufactured by Shunze Mining Machinery Manufacturing Co., Ltd., Changsha, China) for a certain number of minutes to achieve the specific fineness, in accordance with a feed concentration of 65%, to generate an ore slurry. The ground material was further processed using the Davis magnetic tube at an optimized intensity of 0.1 T, resulting in an iron concentrate and a second-stage magnetic tailing. The first-stage and second-stage magnetic tailings were joined to form the final tailing.

The requisite amount of extra calcium oxide (high alkalinity) and coke powder is determined by the fundamental reactions [24] of calcification and magnetization, which can be expressed as follows:

Coke combustion and Boudouard reactions:

2C(s) + O_2_(g) = 2CO(g) (1)

C(s) + O_2_(g) = CO_2_(g) (2)

C(s) + CO_2_(g) = 2CO(g) (3)

Decomposition of goethite and scorodite:

FeO(OH)(s) = Fe_2_O_3_(s) + H_2_O(g) (4)

FeAsO_4_ · 2H_2_O(s) = FeAsO_4_(s) + H_2_O(g) (5)

FeAsO_4_(s) = Fe_2_O_3_(s) + As_2_O_3_(g) + O_2_(g) (6)

Arsenate calcification:

6FeAsO_4_(s) + 9CaO(s) + C(s) = 3Ca_3_(AsO_4_)_2_(s) + 2Fe_3_O_4_(s) + CO(g) (7)

Iron oxides reduction:

3Fe_2_O_3_(s) + C(s) = 2Fe_3_O_4_(s) + CO(g) (8)

Fe_3_O_4_(s) + C(s) = 3FeO (s) + CO(g) (9)

FeO(s) + C(s) = Fe(s) + CO(g)(10)

Through the CMR-LMS process, the segregation of arsenic and iron minerals from iron ores containing arsenic is achieved. This process involves the magnetizing roast method to reduce hematite and goethite to magnetite by coke. The increase in alkalinity allows for the transformation of iron arsenate into a more stable form of calcium arsenate through the reaction (7). Based on the mass fraction 45.32% of total iron, the calculated dosage of external carbon reductant in reaction (8) should be at least 1.63% to convert the high-valence iron oxides in the ore to magnetite. Similarly, the calculated dosage of carbon reductant in reactions (8)–(10) should be at least 14.62% to reduce the high-valence iron oxides in the ore to metallic iron. The usage of 8% coke powder falls within the middle range of this spectrum and is employed for magnetizing roasting to maintain a sufficiently reducing environment, thereby facilitating the formation of highly magnetic magnetite and partial wüstite and metallic iron.

#### 2.2.2. Characterization Test

The particle size distribution of raw ore was tested with the laser particle size analyzer Bettersize 2600. The particle size of the primary magnetic separation concentrate from wet milling is obtained by sieving. A Bruker D8 Advance X-ray diffraction spectroscopy (D8ADVANCE, Bruker, Mannheim, Germany) with Cu Kα radiation was used to determine the mineral phase composition of the raw sample, the roasted sample, and the final magnetic concentrate. SEM and EDX (XL30ESEM-TEP, Philips, Eindhoven, The Netherlands) were employed together to identify the associative properties of minerals in the material, the roasted material, and the magnetic concentrate, as well as to ascertain the elemental distributions in different mineral phases. By carrying out filtration, dehydration, weighing, and chemical analysis of the products obtained from magnetic separation of As and Fe, the metallurgical balances and calculations can be performed to determine the iron recovery rate and arsenic removal rate. The arsenic removal rate and iron recovery were calculated according to Equations (11) and (12), where *ε*, *η*, *γ*, *β*, and *α* represent the arsenic removal, iron recovery, the yield of magnetic separation, the As or Fe grade of the magnetic concentrate, and the As or Fe grade of the raw iron ore, respectively.

(11) $$\varepsilon =\left(1-\gamma \cdot \frac{\beta_{\mathrm{As}}}{\alpha_{\mathrm{As}}}\right)\times 100\%$$

(12) $$\eta =\gamma \cdot \frac{\beta_{\mathrm{Fe}}}{\alpha_{\mathrm{Fe}}}\times 100\%$$

## 3. Results and Discussion

### 3.1. Main Factors of Calcification-Magnetic Roasting

#### 3.1.1. Roasting Temperature

**Figure 5:**
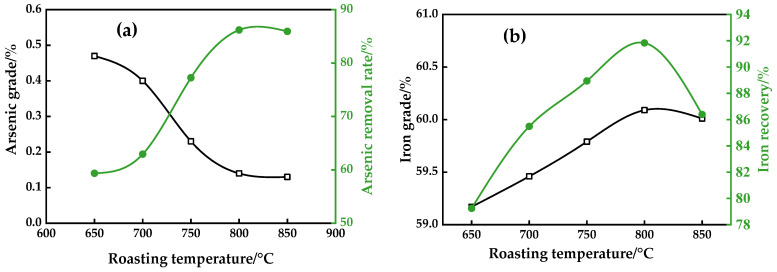
Effect of roasting temperature on arsenic removal (**a**) and iron recovery (**b**).

The results of Figure 5, with a roasting time of 40 min, a dosage of 8% coke, and an alkalinity of 2.42, revealed the influence of the roasting temperature on the arsenic grade of the magnetic concentrate, the arsenic removal rate, the iron grade, and the recovery.

It could be seen from Figure 5 that the increase in roasting temperature from 650 °C to 850 °C had a significant effect on the iron recovery, the arsenic grade of the magnetic concentrate, and the arsenic removal rate, while having a slight effect on the iron grade. As shown in Figure 5a, the increase in the roasting temperature decreased the arsenic content of the magnetic concentrate, and then flattened out at 800 °C. However, the removal rate of arsenic increased initially with the increase in the roasting temperature, and it approached a peak at 800 °C, at which a removal rate of 86.20% was achieved, and then decreased. It could be seen from Figure 5b that the iron grade of the magnetic concentrate changed slightly from 59.17% to 60.09%, and iron recovery initially increased with an increasing roasting temperature, and then decreased after the temperature reached 800 °C. At this temperature, a magnetic concentrate assaying 60.09% Fe with 91.83% recovery was obtained.

The phenomenon could be attributed to the fact that the reaction was incomplete, due to the limited reaction rate at a low temperature. With an increasing roasting temperature, the gasification speed of solid carbon accelerated, and the high CO percentage in the gas phase strengthened the conversion of the iron oxides and ferric arsenates. Parts of the iron oxides were converted to FeO or metallic iron, respectively. Meanwhile, ferric arsenate reacted with CaO and C/CO to form calcium arsenates when the temperature was elevated. This subsequently deteriorated the separation of iron minerals from gangue minerals, and of iron minerals from arsenic minerals, respectively. In the experiment, the target was a high recovery, grade of iron, and removal efficiency of arsenic. Therefore, the roasting temperature around 800 °C was favorable for the recovery of iron and the removal of arsenic.

#### 3.1.2. Roasting Time

**Figure 6:**
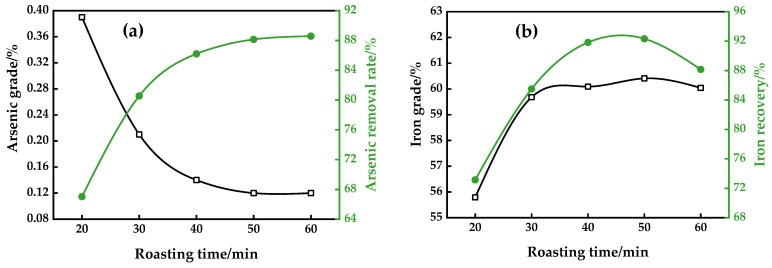
Effect of roasting time on arsenic removal (**a**) and iron recovery (**b**).

Roasting time is a critical factor that influences the arsenic grade of magnetic concentrate, the arsenic removal rate, and the iron grade and recovery. Figure 6 displays the relationship between these variables at a roasting temperature of 800 °C, with a dosage of 8% coke and an alkalinity of 2.42, for different roasting times.

The opposite tendency was observed with the arsenic grade and removal rate, as shown in Figure 6a. The change in the arsenic grade and arsenic removal rate remained relatively stable after 50 min, at which point a magnetic concentrate assaying 0.12% As with 88.14% arsenic removal rate was obtained. It could be seen from Figure 6b that the iron grade and recovery of the magnetic concentrate increased with increasing roasting time, reaching a peak point at 50 min, where a concentrate assaying 64.68% Fe with an 87.89% recovery was obtained. Then, it declined with increasing roasting time.

The reaction rate being limited meant that the reaction was not able to be completed as the roasting time was too short. If the roasting process was prolonged, the high-valence iron oxide could be excessively reduced to ferrous oxide with a diminished magnetism, making it difficult to effectively distinguish iron minerals from the accompanying gangue minerals and calcium arsenates. Therefore, it could be inferred that when the optimum roasting time was set at 50 min, the reduction reactions of hematite to magnetite and the conversion of arsenic in the ferric arsenates to calcium arsenates were mostly completed, resulting in a higher separation efficiency.

#### 3.1.3. Coke Dosage

An analysis was conducted to explore the influence of the reductant dosage on iron grade and recovery, as well as arsenic grade and arsenic removal rate in the magnetic concentrates, when the roasting temperature was set at 800 °C for 50 min with various coke dosages and an alkalinity of 2.42.

**Figure 7:**
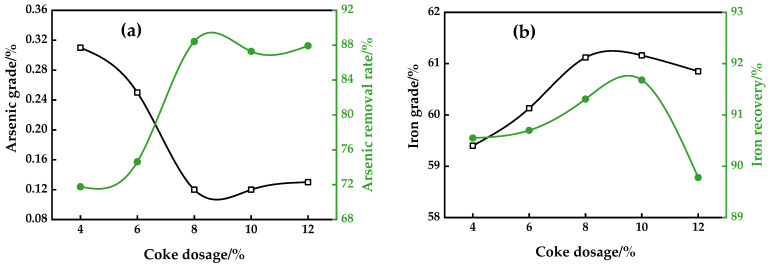
Effect of coke dosage on arsenic removal (**a**) and iron recovery (**b**).

It is evident from Figure 7a that the arsenic grade and arsenic removal rate display an inverse relationship at levels below 8% coke fines, eventually reaching a plateau with an arsenic recovery rate of 88.42% and a grade of 0.12% As. Studies [2] have revealed that there is a specific dosage of reducing agents that must be adhered to; going beyond this value could cause an increase in the emission of arsenic oxides in gas form. Figure 7b demonstrates that the iron grade and recovery was augmented with an increase in the coke dosage, until it attained its maximum value and then decreased when the dosage was approximately 10%. The lower conversion of arsenic-bearing minerals and weakly magnetic iron minerals at a lower dosage of reductants, coupled with the over-reduction in the formed magnetite at a higher dosage, can explain the results observed. This over-reduction subsequently reduces the separation efficiency of the iron minerals.

#### 3.1.4. Alkalinity

**Figure 8:**
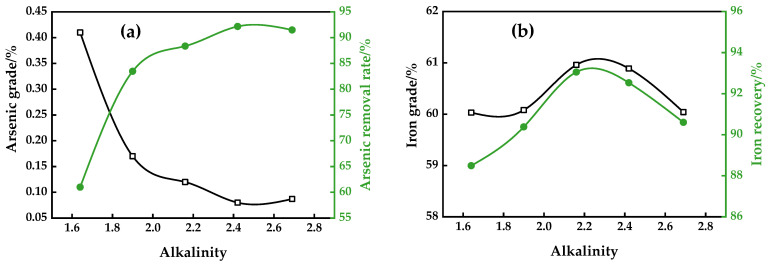
Effect of alkalinity on arsenic removal (**a**) and iron recovery (**b**).

The effects of alkalinity in the CMR-LMS on the separation results were shown in Figure 7. The alkalinity is set to 1.64, 1.90, 2.16, 2.42, and 2.69 by adding an extra CaO dosage from 3–11% with a step length of 2%. As demonstrated in Figure 8, increasing the alkalinity resulted in an increase in the arsenic removal rate until reaching its maximum value and flattening out at an alkalinity of 2.42, where a concentrate containing 0.12% As was created with an iron recovery rate of 92.53%. When the alkalinity ranged from the origin to 2.16, there was a reduced transformation of minerals containing arsenic. The conversion of arsenic-bearing minerals is completed when the alkalinity is above 2.42. Over the extra 9% CaO dosage, it has little effect on the removal of arsenic. The results of the effect of alkalinity are analogous to the result of the dearsenization in iron ore sintering. During high alkaline sintering, a portion of As_2_O_3_ (g) will react with the oxides of Al or Ca to form AlAsO_4_ and Ca_3_(AsO_4_)_2_, thereby leaving residual arsenic in the sinter [3].

According to Figure 8b, the iron grade and recovery rose with an increased alkalinity until they reached their maximum at an alkalinity of 2.16, and then decreased. Meanwhile, the higher alkalinity had a slight effect on the iron grade, increasing it from 60.03% to 60.96%, and a negative effect on the recovery of iron, decreasing it from 88.49% to 93.05%. The result may be attributed to the proper CaO dosage, which could facilitate the conversion of weakly magnetic iron minerals. On the other hand, an excessive CaO could interact with FeO and Fe_2_O_3_ to produce calcium ferrite [25], thus impairing the low-intensity magnetic separation.

### 3.2. Effect of Grinding Fineness of the Second-Staged Magnetic Separation

**Figure 9:**
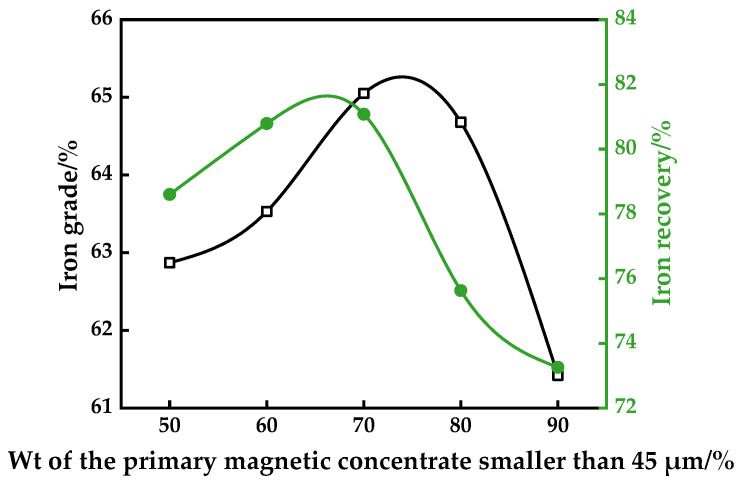
Effect of fineness of the primary magnetic concentrate grinding on the efficacy of the second magnetic separation.

The liberation of gangue minerals from the formed magnetite played a key role in the CMR-LMS process to achieve a high-quality iron concentrate. In this investigation, the effect of feed particle size on the second-stage low-intensity separation results with the CMR-LMS process was tested. The results are illustrated in Figure 9.

The grade and recovery of iron concentrate had the same tendency with the increase in the percentage of less than 45 μm in the feed. They increased until approaching the maximum values of around 70% particle size passing 45 μm, and then decreased. The maximum iron recovery was around 81.08%, with a 65.05% iron grade. This observation may be explained by the higher liberation of magnetite at a finer particle size, leading to a better quality of iron concentrate. The decrease in the recovery was due to the fact that it was more difficult to capture fine magnetite, as opposed to the coarse one, through magnetic separation.

### 3.3. Beneficiation of Arsenic and Iron Minerals with the CMR-LMS Process

**Figure 10:**
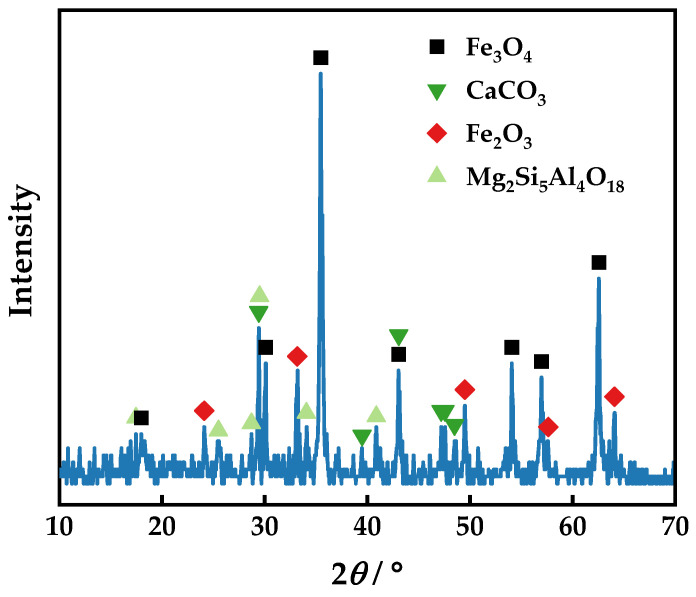
XRD patterns of the sample following roasting and cooling.

**Figure 11:**
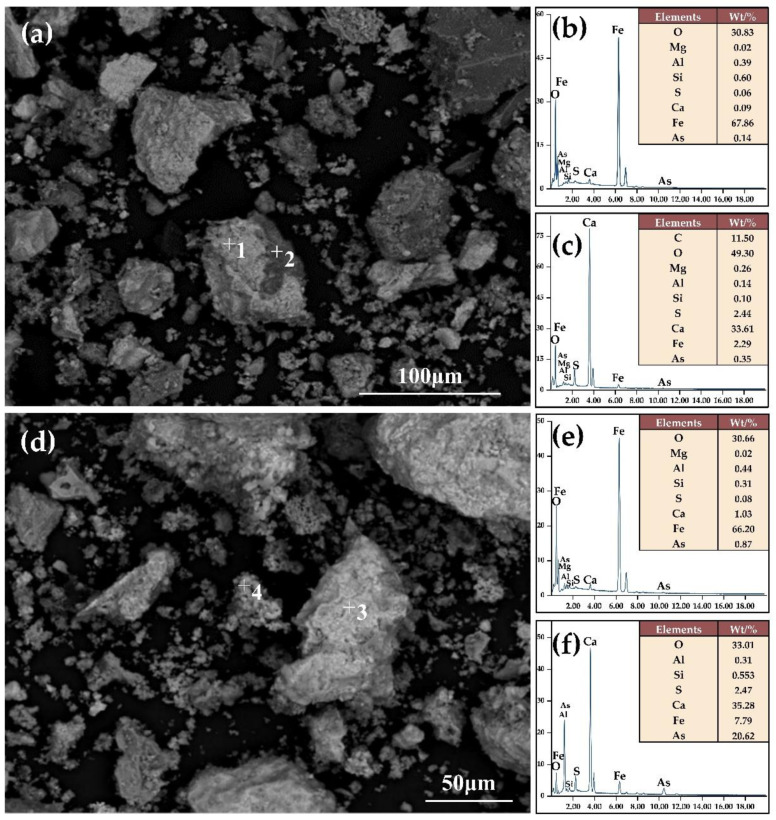
Microscopic morphology (**a**,**d**) of the roasted sample and elements analysis of points 1–4 categorized as (**b**,**c**,**e**,**f**), respectively.

The roasted sample contains magnetic iron minerals that experienced a magnetic reduction. The XRD patterns of the sample roasted at 800 °C for 50 min with 10% coke at an alkalinity of 2.42 are given in Figure 10. The phase analysis of the roasted sample reveals its primary components to be magnetite, calcium carbonate, hematite, and magnesium aluminum silicate, suggesting that hematite and goethite have been transformed into magnetite. The SEM–EDX analysis in Figure 11 reveals that the surface morphology of the sample particles after magnetization roasting is rougher than before, with less than 50 μm having significantly more particles than the original ore. Additionally, the EDX patterns on the right of Figure 11 demonstrate that the newly formed, smaller particles have more calcium, suggesting that gangue composition has been partly stripped from the original ore by magnetization roasting. The result implies that scorodite has been converted into calcium arsenate, which plays a key role in the removal of arsenic. The visible fissures in the roasted particles enable the grinding to effortlessly separate the iron-bearing minerals from the gangue minerals.

**Figure 12:**
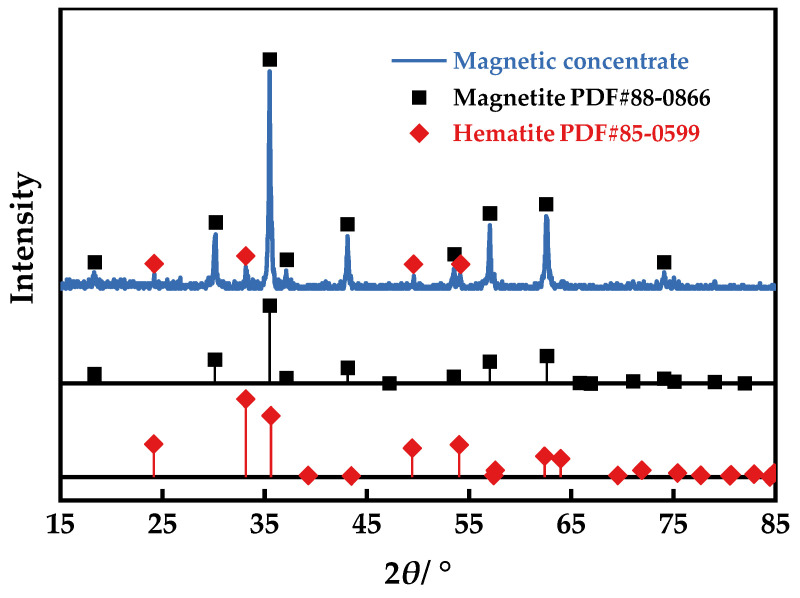
XRD patterns of the final magnetic concentrate.

**Figure 13:**
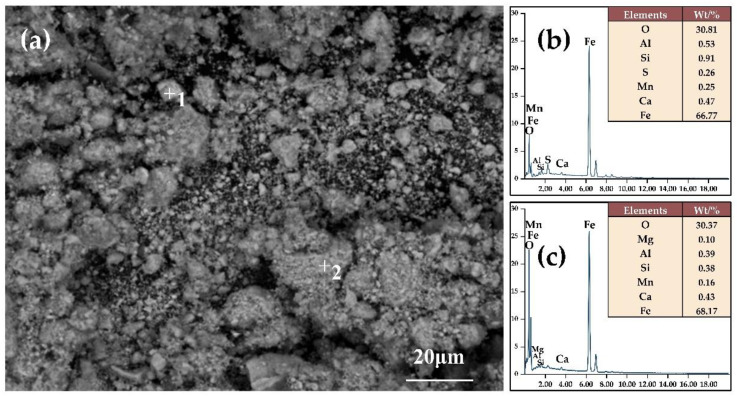
Microscopic morphology (**a**) of the final magnetic concentrate and elements analysis of points 1–2 categorized as (**b**) and (**c**), respectively.

The final iron concentrate that has gone through secondary magnetic separation has a predetermined total iron content. Roasting with 10% coke, an alkalinity of 2.42, and a temperature of 800 °C for 50 min, and with 70% of the particles of the primary magnetic concentrate being smaller than 45 μm after grinding, as indicated in Figure 12, the predominant minerals in the end concentrate are magnetite, accompanied by a smaller proportion of hematite. Results from the EDX point scanning elemental analysis, as demonstrated in Figure 13b,c, indicate that the total iron content was over 65%, which is consistent with the total iron content obtained from chemical analysis in Figure 9. The microscopic morphology of the concentrate, shown in Figure 13a, reveals that the particle size of the iron concentrate is relatively fine, even though the agglomerated large particles are prevalently still smaller than 20 μm.

The results of the magnetic separation process demonstrate that the CMR-LMS process is capable of effectively segregating the magnetic iron minerals from the gangue minerals. The results of the separation, as depicted in Table 4, were obtained under the optimal conditions of roasting the raw ore with 10% coke at an alkalinity of 2.42 at 800 °C for 50 min, and grinding the primary magnetic concentrate to a particle size with 70% of the particles measuring less than 45 μm. The grade and recovery of As/Fe are calculated by two consecutive stages of low intensity magnetic separation. There is a substantial achievement of separation efficiency. An iron concentrate with a 65.65% Fe assay and 80.07% recovery rate was produced through the CMR-LMS process, and the arsenic removal rate was 93.29% with a 0.085% As content. The iron grade of the iron concentrate can be augmented by approximately fourpercentage points when compared to the initial magnetic separation. Despite the decreased Fe recovery due to the finer particle size of the magnetic separation, the iron recovery is still at a high level compared to current reports on magnetization roasting and magnetic separation [4,26].

## 4. Conclusions

The calcification-magnetizing roasting and magnetic separation process for the arsenic-containing iron ore assaying 45.23% Fe and 0.70% As enable a significant separation of the gangue components and arsenic from the iron concentrate. When the high-alkalinity arsenic-containing iron ore is subjected to coke powder roasting, iron arsenate is converted into thermally stable calcium arsenate under the action of the reductant, which is crucial for arsenic removal. Simultaneously, the iron oxides are transformed into magnetic magnetite, allowing for the recovery of iron through weak magnetic separation. The optimum process conditions are roasting with 10% coke, an alkalinity of 2.42, and a temperature of 800 °C for 50 min, and with 70% of the particles of the primary magnetic concentrate being smaller than 45 μm after grinding. Through the CMR-LMS process, a second low-intensity magnetic separation yielded a high-grade magnetic concentrate with 65.65% Fe and 0.085% As, resulting in a 93.29% As removal rate and 80.07% Fe recovery.

## Figures and Tables

**Table 1:** Main chemical composition of the arsenic-bearing iron ore by assaying (mass fraction %).

Element	Fe_total_	As	CaO	MgO	SiO_2_	Al_2_O_3_	S	P	Loss	Total
Contents	45.32	0.70	9.46	3.58	7.62	3.86	0.61	0.06	28.79	100.00

**Table 2:** Distribution of the main iron minerals in arsenic-bearing iron ore (mass fraction %).

Iron Minerals	Magnetite	Siderite	Ferrosilite	Pyrite	Hematite and Limonite	Total
Distribution	4.18	0.29	5.29	4.85	85.39	100.00

**Table 3:** True density and fineness of the arsenic-bearing iron ore.

Physical Property	True Density/(g/cm^3^)	Blain Specific Surface Area/(cm^2^/g)	Grain Size Mode/μm
Arsenic-bearing iron ore	3.79	603.8	105.4
Standard cement sample	3.18	3870	-

**Table 4:** Beneficiation results of the sample with the CMR-LMS process (unit %).

Product	Yield	Grade		Recovery	
		As	Fe	As	Fe
Magnetic concentrate	55.27	0.085	65.65	6.71	80.06
Tailings	44.73	1.46	20.20	93.29	19.94
Feed	100.00	100.00	45.32	100.00	100.00

## Data Availability

The data that support the findings of this study are available from the corresponding author, Y.Z., upon reasonable request.
